# Supplementary material for: The interactome of intact mitochondria by cross-linking mass spectrometry provides evidence for coexisting respiratory supercomplexes
Source: Mol Cell Proteomics. 2017 Dec 8;17(2):216–32. doi: 10.1074/mcp.RA117.000470 (PMC5795388; doi:10.1074/mcp.RA117.000470)
Supplement: Supplemental Data [file supp_RA117.000470_133922_0_supp_23978_fzffwf.zip › spectra_annotation/mito_spectra_annotation/268-1-4-1-8-1.pdf]

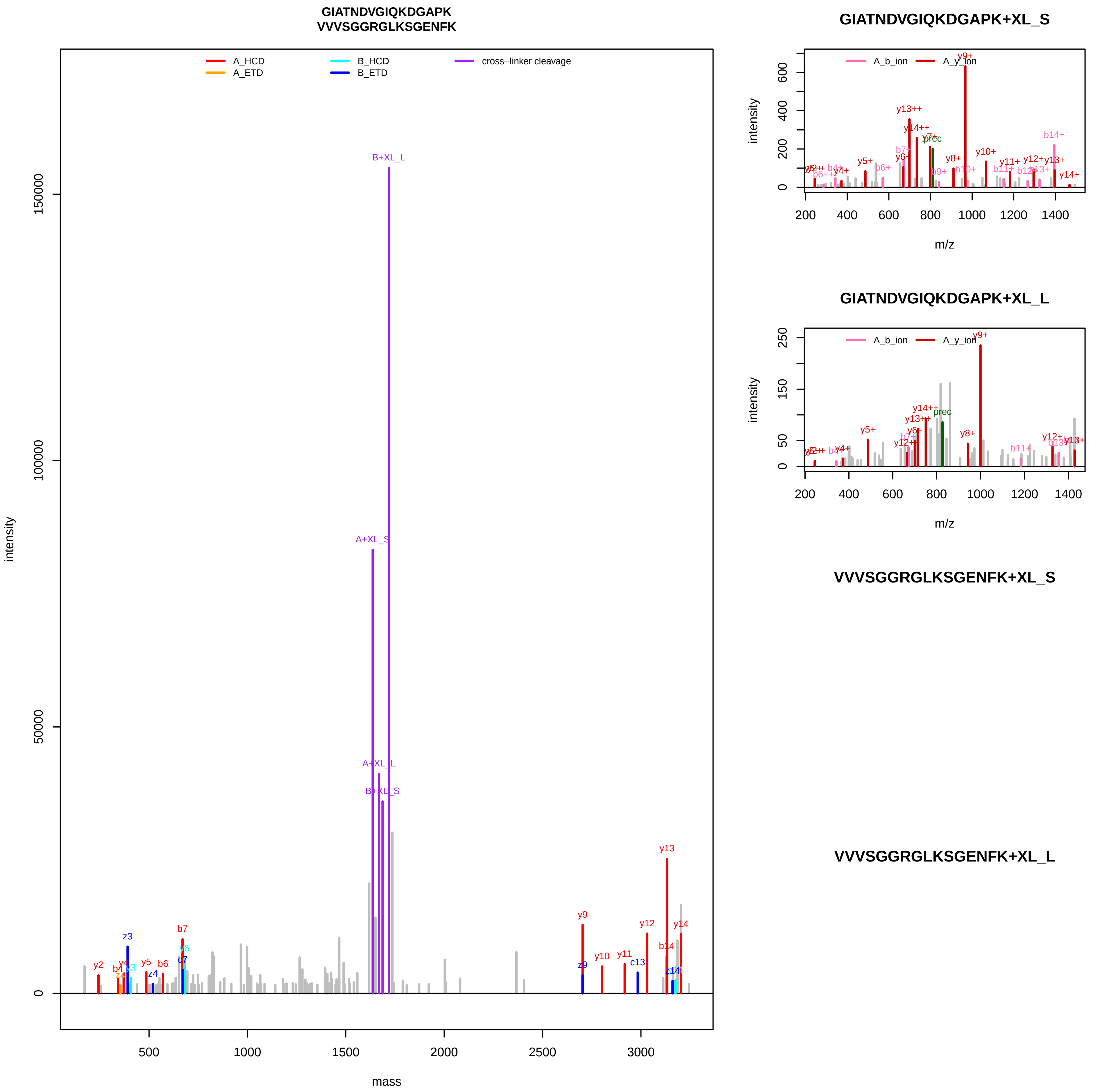
GIATNDVGIQKDGAPK
VVVSGGRGLKSGENFK
A_HCD
A_ETD
B_HCD
B_ETD
cross−linker cleavage
B+XL_L
A+XL_S
A+XL_L
B+XL_S
y2
b4
y4
z3
y3
z4
y5
b6
b7
y6
c7
y9
z9
y10
y11
c13
y12
b14
y13
y14
z14
intensity
mass
0
50000
100000
150000
500
1000
1500
2000
2500
3000
GIATNDVGIQKDGAPK+XL_S
A_b_ion
A_y_ion
y9+
y13++
y14++
y7+
prec
b14+
y10+
y8+
y11+
y12+
y13+
y14+
y5+
y6+
b7+
b6+
y4+
b4+
b10+
b9+
b11+
b12+
b13+
intensity
m/z
0
200
400
600
200
400
600
800
1000
1200
1400
GIATNDVGIQKDGAPK+XL_L
A_b_ion
A_y_ion
y9+
y14++
y13++
prec
y5+
y6+
y8+
y12+
b7+
y4+
b4+
b11+
y12+
b13+
y13+
intensity
m/z
0
50
150
250
200
400
600
800
1000
1200
1400
VVVSGGRGLKSGENFK+XL_S
VVVSGGRGLKSGENFK+XL_L
